# Growth, Root, Photosynthetic, and Soil Responses of Poplar Seedlings to an Equal-Mass PVC–PE–PS Microplastic Mixture

**DOI:** 10.3390/biology15171534

**Published:** 2026-09-04

**Authors:** Jun Dong, Jinbo Li, Jinlong Li, Zimo Zhang, Nan Xu, Haixiu Zhong, Lijun Zhou

**Affiliations:** 1 Heilongjiang Province Key Laboratory of Cold Region Wetland Ecology and Environment Research, Harbin University, Harbin 150086, China; 2 Heilongjiang Academy of Sciences Institute of Natural Resources and Ecology, Harbin 150040, China; 3 College of Agricultural and Hydraulic Engineering, Suihua University, Suihua 152061, China

**Keywords:** conventional microplastics, polyvinyl chloride, polyethylene, polystyrene, poplar seedlings, root architecture, photosynthesis, oxidative injury, biomass, antioxidant enzyme activity

## Abstract

Soil microplastic pollution may affect woody plant establishment, but evidence for tree seedlings remains limited. Populus simonii × P. nigra ‘1307’ seedlings were grown for 45 d in soil containing an equal-mass PVC–PE–PS mixture at 0, 100, 500, or 1000 mg kg^−1^ dry soil. Growth, root architecture and activity, photosynthetic traits, oxidative status, and soil chemical and enzymatic properties were measured. The 100 mg kg^−1^ treatment produced limited, trait-specific changes and did not consistently inhibit growth. In contrast, 500 and 1000 mg kg^−1^ reduced most growth and physiological traits. At 1000 mg kg^−1^, total dry biomass, total root length, root surface area, root volume, root-tip number, and root activity decreased by 43.7%, 46.5%, 47.2%, 50.2%, 51.1%, and 50.9%, respectively. Net photosynthetic rate and PSII electron transport declined, whereas H_2_O_2_, O_2_•^−^ production, thiobarbituric acid-reactive substances (TBARS), and electrolyte leakage increased. Under higher exposure, soil electrical conductivity was higher, whereas available nutrient levels and several soil enzyme activities were lower. These results indicate that medium and high concentrations of the tested mixture were associated with concurrent inhibition of root development, photosynthetic performance, and biomass accumulation under short-term pot conditions. Because concurrent impairment of woody-seedling performance and soil biochemical functioning may compromise vegetation establishment, these findings support the inclusion of mixed-polymer exposure in ecological risk assessments for soils used in forestry and ecological restoration.

## Simple Summary

Microplastic residues are increasingly detected in terrestrial soils, but their effects on woody seedlings remain less studied than their effects on agricultural crops. In this study, poplar seedlings were exposed for 45 d to an equal-mass mixture of polyvinyl chloride, polyethylene, and polystyrene microplastics at 0, 100, 500, and 1000 mg kg^−1^ dry soil. The lowest exposure was associated with limited and trait-specific changes, without consistent growth inhibition. In contrast, the two higher exposure treatments were associated with reduced root development, biomass accumulation, photosynthetic pigments, gas exchange, and photosystem II performance, together with greater oxidative injury and changes in soil nutrient availability and enzyme activities. These findings show that medium and high concentrations of the tested microplastic mixture were associated with concurrent changes in soil, root, and leaf traits. Longer-term studies are required to determine whether these responses also occur under field conditions.

## 1. Introduction

Microplastics are commonly defined as plastic particles smaller than 5 mm and are increasingly recognized as contaminants of terrestrial ecosystems [1,2,3]. Agricultural soils can receive microplastics from plastic mulch, greenhouse films, irrigation materials, sewage sludge, compost, atmospheric deposition, and the fragmentation of larger plastic residues [4,5]. After becoming part of soil, microplastics can alter aggregation, porosity, water flow, aeration, availability of nutrients, and microbial action. The changes may influence the development of roots and plant growth but the orientation and extent of the response will vary with the type of polymer, the size of the particle, the concentration of the polymer, soil properties, the time of exposure, and the type of plant [6,7,8,9].

Polyvinyl chloride (PVC), polyethylene (PE), and polystyrene (PS) are widely used in agricultural films, greenhouse materials, irrigation components, packaging, and other products, and residues of different polymers may coexist in terrestrial soils. Most previous plant studies have examined individual polymers or compared polymers in separate treatments. Recent terrestrial studies have extended this work by evaluating polymer-specific effects on soil-rhizosphere-plant systems, plant–soil feedbacks, environmentally oriented soil–plant exposure, and mixed-polymer effects in terrestrial mesocosms [10,11,12,13]. However, these studies have mainly focused on crops, herbaceous species, soil microbial processes, or polymer formulations different from those examined here. Evidence remains limited for woody seedlings exposed to a controlled mixture of conventional polymers.

In particular, few studies have simultaneously examined root architecture and activity, photosynthetic pigments, gas exchange, PSII photochemistry, oxidative status, antioxidant responses, whole-plant biomass, and root-zone soil biochemical properties under the same mixed-polymer formulation. The present study addresses this gap by evaluating concentration-dependent responses of poplar seedlings and their root-zone soil to a fixed equal-mass PVC–PE–PS mixture. The novelty of the study lies in its integrated assessment of concurrent soil, root, leaf, and whole-plant responses within one controlled experimental framework. Because single-polymer treatments were not included, the study was not designed to distinguish polymer-specific effects or determine whether the combined responses were additive, synergistic, or antagonistic.

The roots are the primary interaction between plants and soil with microplastics. Modifications of the physical structure of the soil, the movement of nutrients, rhizosphere processes, or touching the roots with particles can cause changes in root elongation, branching, absorptive surface area, and metabolic activity [6,8]. The limited root development could then be accompanied by a smaller water and nutrient uptake and diminished shoot growth. Root architecture and root activity are thus relevant endpoints to assess plant response to soil microplastics.

Photosynthetic properties are used to give supplementary information concerning the aboveground responses. The net photosynthetic rate, stomatal conductance, intercellular CO_2_ concentration and transpiration rate can be applied to assess restrictions imposed on the process of carbon assimilation [14,15]. Chlorophyll fluorescence parameters such as Fv/Fm, Y(II), electron transport rate, non-photochemical quenching, and photochemical quenching have been used to explain various features of the efficiency of photosystem II, electron transport, heat dissipation, and the openness of the reaction centers [16,17,18]. Gas-exchange and fluorescence measurements may assist in discriminating between those caused by stomatal control and those caused by photochemical or biochemical factors.

Microplastic exposure has also been shown to affect the cellular redox balance. Constraints on root activities, mineral uptake, carbon fixation, or photosynthetic electron transfer can be accompanied by increased formation of reactive oxygen species. Overproduction of H_2_O_2_ and superoxide-anion can cause lipid peroxidation and membrane leakage. In response to these phenomena, plants may alter the activities of superoxide dismutase, peroxidase, catalase, and ascorbate peroxidase, but the increase in enzyme activity is not always able to inhibit oxidative injury [19,20,21].

The chemical and enzymatic endpoints of the soil are also valid. Nitrogen transformation, carbon turnover, oxidative reactions, and phosphorus cycling are related to urease, sucrase, catalase and alkaline phosphatase activities, respectively [22,23]. These activities can be used as an indicator of change in soil biological functionality. Nonetheless, soil and plant reactions recorded at the same endpoint cannot on their own determine whether variations in soil characteristics occurred before plant damage or were partially affected by plant and rhizosphere feedbacks.

Poplar (*Populus* spp.) is an important group of fast-growing woody plants widely used in timber production, shelterbelt establishment, ecological restoration, and biomass production. The hybrid *Populus simonii* × *P. nigra* ‘1307’ used in the present study has also been employed in controlled studies of growth, photosynthetic physiology, chlorophyll fluorescence, and environmental-stress responses. Early root development is particularly important for the establishment of poplar seedlings because it determines their capacity for soil exploration and water and nutrient acquisition and is closely associated with subsequent aboveground growth and photosynthetic performance. Despite the ecological and silvicultural importance of poplar, information remains limited regarding the coordinated soil, root, photosynthetic, oxidative, and growth responses of poplar seedlings to mixed-polymer contamination.

This initial controlled experiment deliberately used one poplar hybrid and one soil type to minimize genetic and edaphic variation. A fixed 1:1:1 PVC–PE–PS formulation was applied across a defined total-concentration gradient so that exposure responses could be evaluated while mixture composition remained constant. The 45 d exposure period was selected to examine responses during the early seedling-establishment stage. Accordingly, the experiment was designed to identify coordinated short-term response patterns rather than to compare poplar genotypes, soil types, mixture ratios, or long-term field responses.

The present study therefore evaluated poplar seedlings exposed to an equal-mass PVC–PE–PS microplastic mixture at four total concentrations. We examined whether increasing exposure was associated with changes in (1) seedling growth and biomass allocation; (2) root architecture and activity; (3) photosynthetic pigments, gas exchange, and PSII performance; (4) oxidative injury and antioxidant enzyme activities; and (5) soil chemical properties and enzyme activities. We hypothesized that medium and high exposure would impair root development, photosynthetic performance, and biomass accumulation and would be associated with greater oxidative injury and less favorable soil biochemical functioning. Responses at the lowest concentration were treated as exploratory because low-dose effects have varied across polymers, soils, and plant species in previous studies.

## 2. Materials and Methods

### 2.1. Plant Material and Growth Conditions

Uniform rooted cuttings of *Populus simonii* × *P. nigra* ‘1307’ were used in this experiment. This hybrid has previously been used in controlled-environment studies of growth, photosynthetic physiology, chlorophyll fluorescence, and stress responses in poplar seedlings [24,25].

Semi-hardwood cuttings approximately 15 cm long and 0.8 cm in diameter were treated with 0.5% indole-3-butyric acid before rooting. After 30 d, rooted seedlings with similar shoot height, stem diameter, leaf number, and root development were selected. At the beginning of the experiment, the selected seedlings had an average height of 30.0 ± 2.5 cm and an average stem diameter of 5.0 ± 0.5 mm.

The experimental soil was a sandy loam collected from the 0–20 cm surface layer. Before use, the soil was air-dried, homogenized, and passed through a 2 mm sieve. Each seedling was transplanted into a plastic pot with a top diameter of 26 cm, a bottom diameter of 21 cm, and a height of 28 cm. Each pot contained 5.0 kg of air-dried soil.

The experiment was conducted beginning in June 2025 in a controlled greenhouse at the Institute of Natural Resources and Ecology, Heilongjiang Academy of Sciences, Harbin, China. The greenhouse was maintained under a 14 h/10 h light/dark photoperiod, day/night temperatures of 25/18 °C, relative humidity of 65–75%, and a photosynthetic photon flux density of approximately 250 μmol m^−2^ s^−1^. Soil moisture was maintained at approximately 60% of the maximum water-holding capacity by weighing the pots and replenishing water loss every 2 d.

### 2.2. Mixed Microplastic Materials and Experimental Design

Commercial polyvinyl chloride (PVC), polyethylene (PE), and polystyrene (PS) microplastic powders were purchased from Wuxi Rigor Biotechnology Co., Ltd. (RIGOR, Wuxi, China). According to the supplier information, the three polymers were supplied as 1000-mesh powders with a nominal particle size of approximately 13 μm. The microplastic particles were used as received and were not further characterized after incorporation into the soil.

Equal dry masses of PVC, PE, and PS were weighed separately and thoroughly combined at a mass ratio of 1:1:1. The resulting material is hereafter referred to as the PVC–PE–PS mixture. This equal-mass formulation was used as a controlled experimental mixture of three conventional polymers. It was not intended to reproduce the precise polymer composition of a specific field soil or to distinguish the individual effects of PVC, PE, and PS.

The 1:1:1 mass ratio was selected as a standardized formulation that provided an equal mass contribution from each polymer and allowed the total concentration of the mixture to be varied while keeping its composition constant. This formulation was used to examine the overall concentration-dependent responses of poplar seedlings and root-zone soil to a controlled mixed-polymer exposure. Because single-polymer treatments were not included, the experiment was not designed to distinguish the individual effects of PVC, PE, and PS or to determine whether their combined effects were additive, synergistic, or antagonistic.

Four total concentrations of the PVC–PE–PS mixture were established: 0 mg kg^−1^ dry soil (CK), 100 mg kg^−1^ dry soil (MP-L), 500 mg kg^−1^ dry soil (MP-M), and 1000 mg kg^−1^ dry soil (MP-H). Because each pot contained 5.0 kg of dry soil, the corresponding amounts of mixed microplastics added to each pot were 0, 0.50, 2.50, and 5.00 g, respectively.

The concentrations of 100, 500, and 1000 mg kg^−1^ dry soil were selected as low, intermediate, and high experimental exposure levels, respectively. Mass-based field surveys have reported estimated microplastic concentrations of approximately 3.12–7.99 mg kg^−1^ in plastic-mulched agricultural soils from different regions of China, while concentrations ranging from 0.85 to 67.12 mg kg^−1^ have been estimated in intensively mulched agricultural soils in Xinjiang [26,27]. A recent field-oriented soil–plant experiment also used PE concentrations of 100 and 600 mg kg^−1^ [28]. Therefore, the 100 mg kg^−1^ treatment was close to or slightly above the upper range of some reported mass-based field measurements, whereas 500 and 1000 mg kg^−1^ represented elevated experimental exposures intended to characterize concentration-dependent responses. Direct comparisons with field concentrations remain approximate because reported values vary with land use, polymer composition, particle size, weathering state, sampling design, and analytical method, and many environmental surveys report particle abundance rather than mass. The selected treatments should therefore be interpreted as a controlled exposure gradient rather than as universal background concentrations in agricultural or forest soils.

For each treatment, the required amount of the PVC–PE–PS mixture was first blended with a small portion of dry soil and then progressively mixed with the remaining soil until no visible concentration gradients remained. Rooted seedlings were acclimated to the greenhouse conditions for 10 d before transplantation into the treated soils. The 45 d exposure period began on the day of transplantation.

Every treatment had six biological replicates. An experimental unit consisted of a single pot with one seedling. The pots were placed randomly around the greenhouse and they were reorganized at 2 days interval to reduce the positional effect. At harvest, growth, biomass, root architecture, root activity, photosynthetic traits, oxidative-status indicators, antioxidant enzyme activities, soil chemical properties, and soil enzyme activities were measured.

### 2.3. Growth Traits and Biomass

Plant height was measured as the distance between the soil surface and the apex of the shoot with a ruler at harvest. The stem diameter was measured with a digital caliper (15 cm above the ground) and the quantity of the fully developed leaves was determined. Loose leaves were scanned in an EPSON DS-60000 scanner (Seiko Epson Corporation, Suwa, Japan) and the total leaf area per plant was measured with ImageJ 1.54p (National Institutes of Health, Bethesda, MD, USA). Shoots and roots were separated. Roots were washed with tap water, rinsed with deionized water, and gently blotted with absorbent paper before fresh mass was recorded. Plant samples were heated at 105 °C for 30 min and then dried at 75 °C to constant mass. Total dry biomass was calculated as the sum of shoot and root dry mass. The root-to-shoot ratio was calculated separately for each seedling as root dry mass divided by shoot dry mass.

### 2.4. Root Architecture Analysis and Root Activity Assay

The roots were carefully cleaned with tap water and after that, they were rinsed with deionized water. The root system was spread evenly in a transparent tray and scanned using a MICROTEK ScanMaker i800 Plus root scanner (Microtek International Inc., Shanghai, China). Root images were analyzed using WinRHIZO 2025a (Regent Instruments Inc., Quebec City, QC, Canada). Total root length, root surface area, root volume, average root diameter, and root-tip number were determined. Root image analysis was used because these variables provide complementary information on root system development, soil exploration, and potential absorptive capacity under stress conditions.

Root activity was determined using the triphenyl tetrazolium chloride (TTC) reduction method. The reduced product, triphenyl formazan (TPF), was extracted with ethyl acetate, and its absorbance was measured at 485 nm using a UV–visible spectrophotometer. Root activity was expressed as μg TPF g^−1^ FW h^−1^.

### 2.5. Photosynthetic Pigments and SPAD Values

At the end of the 45 d exposure period, SPAD values were measured once on fully expanded functional leaves at comparable developmental positions on each seedling using a SPAD-502 Plus chlorophyll meter (Konica Minolta, Tokyo, Japan). Five consecutive readings were taken from different positions of the leaf blade while avoiding the main veins, and the mean of the five readings was used as the value for each biological replicate.

Photosynthetic pigments were extracted from 0.20 g of fresh leaf tissue using 20 mL of 80% acetone in darkness for 24 h. The extract was centrifuged at 10,000× *g* for 10 min. Absorbance was measured at 663, 646, and 470 nm using a UV−1800 UV–visible spectrophotometer (Shimadzu, Kyoto, Japan). Chlorophyll a, chlorophyll b, and carotenoid contents were calculated according to Lichtenthaler and expressed as mg g^−1^ FW [29].

### 2.6. Gas-Exchange Measurements

Leaf gas-exchange parameters were measured between 09:00 and 11:30 using a CIRAS-3 portable photosynthesis system (PP Systems, Amesbury, MA, USA). This morning measurement period was selected to maintain relatively stable physiological conditions and to avoid possible midday depression of photosynthesis. Because irradiance, CO_2_ concentration, leaf temperature, and relative humidity were controlled within the leaf chamber, the measurements were not dependent on ambient full-sun conditions. A fully expanded functional leaf at a comparable developmental position was selected from each seedling. The chamber conditions were maintained at a photosynthetic photon flux density of 1000 μmol m^−2^ s^−1^, a reference CO_2_ concentration of 400 μmol mol^−1^, a leaf temperature of 25 °C, and a relative humidity of 60–70%.

Measurements were recorded after the gas-exchange parameters had stabilized. Net photosynthetic rate (Pn), stomatal conductance (Gs), intercellular CO_2_ concentration (Ci), and transpiration rate (Tr) were recorded. Instantaneous water-use efficiency (WUE) was calculated separately for each biological replicate as WUE = Pn/Tr, where Pn is the net photosynthetic rate and Tr is the transpiration rate. WUE was expressed as μmol CO_2_ mmol^−1^ H_2_O.

### 2.7. Chlorophyll Fluorescence Measurements

Chlorophyll fluorescence parameters were measured using an FMS-2 pulse-modulated chlorophyll fluorometer (Hansatech Instruments Ltd., Norfolk, UK). Leaves were dark-adapted for 30 min before measurement. Minimum fluorescence (F_o_) was recorded under weak measuring light, and maximum fluorescence (Fm) was induced using a saturating pulse. The maximum quantum efficiency of photosystem II was calculated as Fv/Fm = (Fm − Fo)/Fm.

The leaves were subsequently irradiated with actinic light at 600 μmol m^−2^ s^−1^ during 5 min. The steady-state fluorescence (Fs) and the light-adapted maximum fluorescence (Fm) was measured. The effective quantum yield of PSII [Y(II)], electron transport rate (ETR), non-photochemical quenching (NPQ) and the photochemical quenching coefficient (qP) were evaluated by conventional methods [16,17,18,30]. The ETR was determined as Y(II) × PPFD × 0.84 × 0.5.

### 2.8. Oxidative-Injury Indicators and Antioxidant Enzyme Assays

Immediately after the physiological measurements, fresh leaves were collected, frozen in liquid nitrogen, and stored at −80 °C until analysis. For each oxidative-status assay, approximately 0.50 g of fresh leaf tissue was used. Hydrogen peroxide (H_2_O_2_) content was determined according to Patterson et al. and expressed as μmol H_2_O_2_ g^−1^ FW. The superoxide-anion (O_2_•^−^) production rate was determined according to Elstner and Heupel and expressed as nmol O_2_•^−^ g^−1^ FW min^−1^ [31,32]. Thiobarbituric acid-reactive substances (TBARS) were determined according to Heath and Packer and expressed as nmol MDA equivalents g^−1^ FW [33]. Because the colorimetric thiobarbituric acid assay is not specific for MDA in plant tissues, the results were reported as TBARS rather than MDA content [34].

Leaf disks were immersed in deionized water for 12 h at room temperature, and the initial electrical conductivity (C1) was measured. The samples were then boiled for 20 min, cooled to room temperature, and measured again to obtain the total electrical conductivity (C2). Relative electrolyte leakage was calculated as (C1/C2) × 100.

For antioxidant enzyme extraction, 0.50 g of fresh leaf tissue was homogenized in 5 mL of ice-cold 50 mM potassium phosphate buffer (pH 7.0) containing 1 mM EDTA and 1% polyvinylpyrrolidone. The homogenate was centrifuged at 12,000× *g* for 15 min at 4 °C, and the supernatant was used for enzyme assays. Superoxide dismutase (SOD; EC 1.15.1.1) activity was determined by measuring the inhibition of nitroblue tetrazolium photoreduction at 560 nm according to Beauchamp and Fridovich [35]. Peroxidase (POD; EC 1.11.1.7) activity was determined using the guaiacol–H_2_O_2_ method at 470 nm. Catalase (CAT; EC 1.11.1.6) activity was determined by monitoring the decrease in H_2_O_2_ absorbance at 240 nm. Ascorbate peroxidase (APX; EC 1.11.1.11) activity was determined by monitoring the decrease in absorbance at 290 nm due to ascorbate oxidation. POD and CAT activities were determined according to Chance and Maehly, and APX activity was determined according to Nakano and Asada. Enzyme activities were expressed as U g^−1^ FW [36,37].

### 2.9. Root-Zone Soil Chemical Properties

Root-zone soil was collected from each pot at harvest after visible roots and plant debris had been removed. The samples were divided into two portions. One portion was stored at 4 °C for soil enzyme assays, whereas the other was air-dried, ground, and passed through a 2 mm sieve before chemical analysis.

Soil pH was measured in a 1:5 soil-to-water suspension using a calibrated pH meter. Soil electrical conductivity was measured in a 1:5 soil-to-water extract using a calibrated conductivity meter and expressed as μS cm^−1^. Soil organic matter was determined using the K_2_Cr_2_O_7_ oxidation–external heating method and expressed as g kg^−1^. Available nitrogen was determined using the alkaline hydrolysis–diffusion method. Available phosphorus was extracted with NaHCO_3_ and measured using the molybdenum–antimony colorimetric method. Available potassium was extracted with neutral ammonium acetate and determined by flame photometry. Available nutrient contents were expressed as mg kg^−1^ dry soil.

### 2.10. Soil Enzyme Activities

Soil urease activity was determined by incubating soil with urea and colorimetrically quantifying the released ammonium following Kandeler and Gerber [22]. Activity was expressed as U g^−1^ soil according to the unit definition used in the analytical procedure.

Soil sucrase activity was determined by quantifying the glucose released from sucrose and expressed as mg glucose g^−1^ soil 24 h^−1^. Soil catalase activity was determined from the decomposition of H_2_O_2_ and expressed as mL 0.02 M KMnO_4_ g^−1^ soil h^−1^. Alkaline phosphatase activity was determined using disodium phenyl phosphate as the substrate and expressed as mg phenol g^−1^ soil 24 h^−1^. The soil-enzyme assays followed the general procedures described by Kandeler and Gerber and Tabatabai [22,23].

Soil microbial biomass and community composition were not measured in the present study; therefore, the measured enzyme activities were interpreted as soil biochemical response indicators rather than direct measures of microbial activity.

### 2.11. Statistical Analysis

Data are presented as means ± standard deviations (SD) based on six biological replicates per treatment. One pot containing one seedling was considered one independent experimental unit. Differences among CK, MP-L, MP-M, and MP-H were evaluated using one-way analysis of variance followed by Tukey’s honestly significant difference test. Statistical significance was defined as *p* < 0.05. All derived variables were calculated separately for each biological replicate before statistical analysis. Complete one-way ANOVA statistics for all measured variables, including degrees of freedom, F values, *p* values, partial η^2^, and Tukey HSD grouping information, are provided in Supplementary Table S1. Figures were prepared using OriginPro 2024 (OriginLab Corporation, Northampton, MA, USA).

## 3. Results

### 3.1. Growth and Biomass Responses to the PVC–PE–PS Mixture

**Figure 1:**
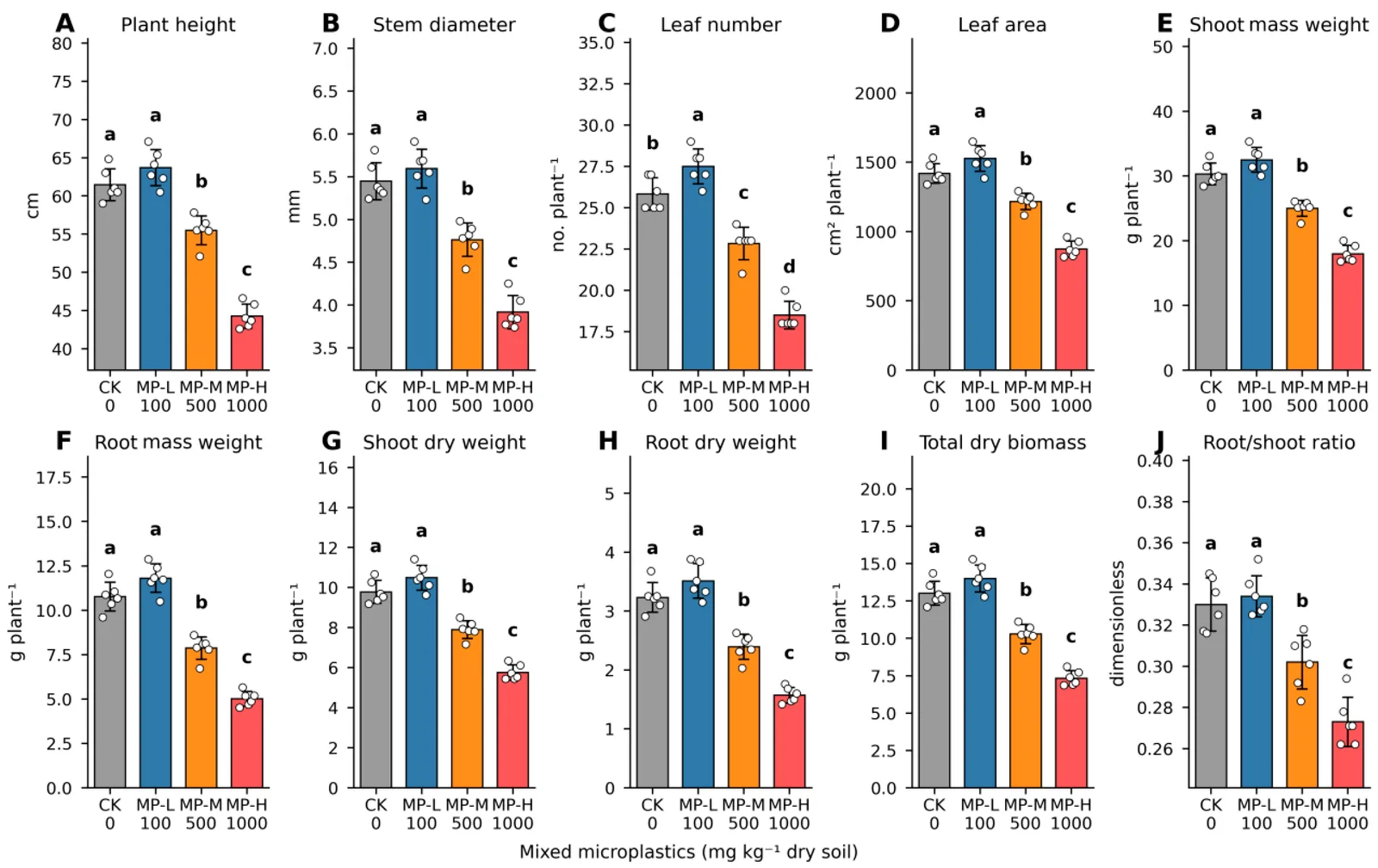
Effects of an equal-mass PVC–PE–PS microplastic mixture on the growth and biomass of poplar seedlings: (**A**) plant height; (**B**) stem diameter; (**C**) leaf number; (**D**) total leaf area; (**E**) shoot fresh mass; (**F**) root fresh mass; (**G**) shoot dry mass; (**H**) root dry mass; (**I**) total dry biomass; and (**J**) root-to-shoot ratio. CK, MP-L, MP-M, and MP-H represent total mixture concentrations of 0, 100, 500, and 1000 mg kg^−1^ dry soil, respectively. Values are means ± SD (*n* = 6). Different lowercase letters indicate significant differences among treatments according to Tukey’s HSD test at *p* < 0.05.

Growth and biomass were significantly reduced under MP-M and MP-H, with the strongest inhibition observed under MP-H. Compared with CK, MP-H reduced plant height, stem diameter, leaf number, leaf area, shoot fresh mass, root fresh mass, shoot dry mass, root dry mass, and total dry biomass by 28.0%, 28.1%, 28.4%, 38.4%, 40.7%, 53.5%, 41.1%, 51.4%, and 43.7%, respectively (Figure 1A–I). MP-M also reduced these traits, although the reductions were smaller than those observed under MP-H. The root-to-shoot ratio decreased from 0.33 in CK to 0.30 in MP-M and 0.27 in MP-H (Figure 1J), reflecting a proportionally greater reduction in root dry mass than in shoot dry mass at the two higher exposure levels. In contrast, MP-L did not significantly affect plant height, stem diameter, leaf area, shoot or root fresh mass, shoot or root dry mass, total dry biomass, or the root-to-shoot ratio, whereas leaf number was significantly higher than that in CK. Full one-way ANOVA results for all measured variables are provided in Supplementary Table S1.

### 3.2. Root Architecture and Root Activity

**Figure 2:**
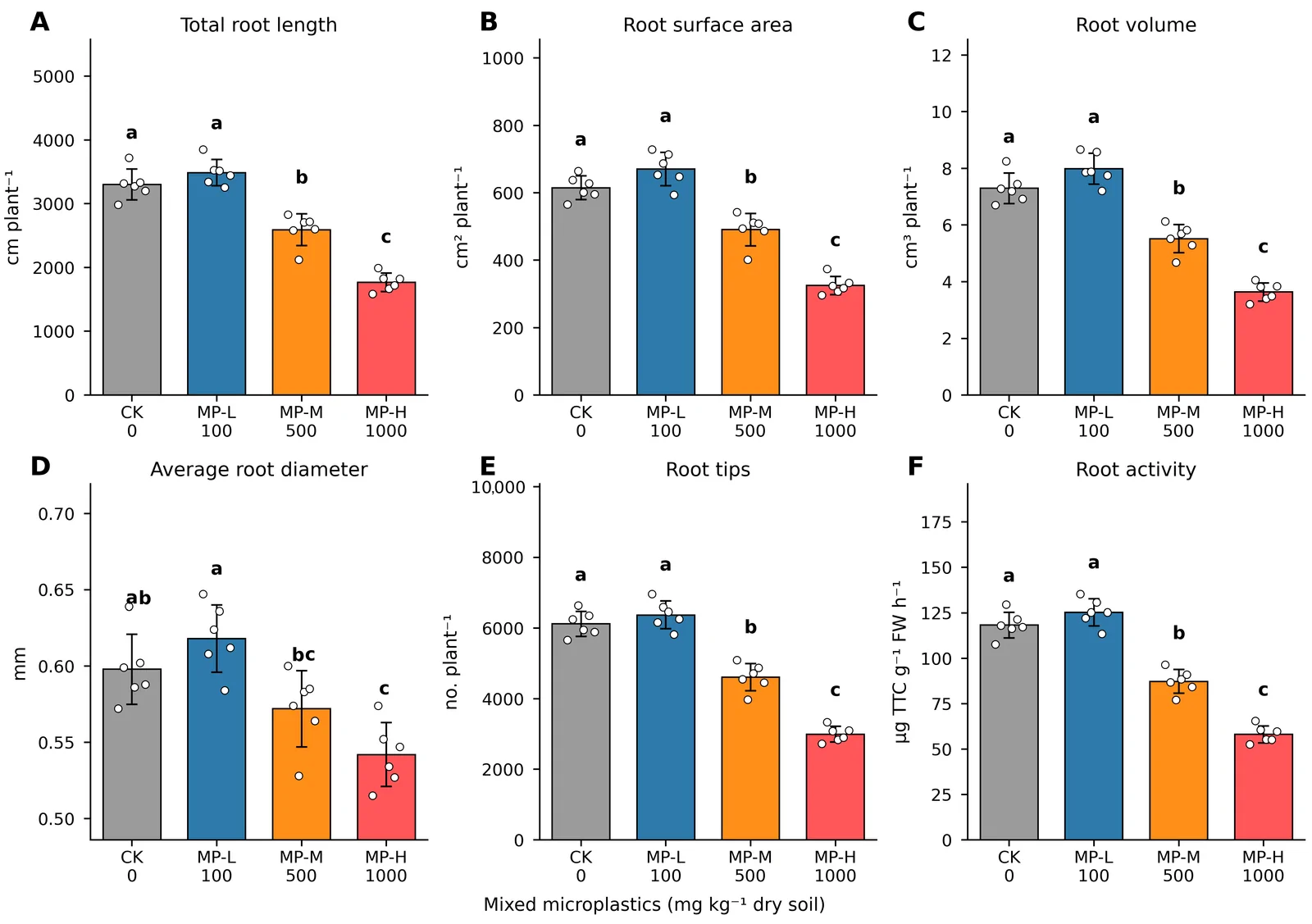
Effects of an equal-mass PVC–PE–PS microplastic mixture on the root architecture and root activity of poplar seedlings: (**A**) total root length; (**B**) root surface area; (**C**) root volume; (**D**) average root diameter; (**E**) root-tip number; and (**F**) root activity. CK, MP-L, MP-M, and MP-H represent total mixture concentrations of 0, 100, 500, and 1000 mg kg^−1^ dry soil, respectively. Values are means ± SD (*n* = 6). Different lowercase letters indicate significant differences among treatments according to Tukey’s HSD test at *p* < 0.05.

Root development was significantly inhibited under MP-M and MP-H, with the strongest effects observed under MP-H. Compared with CK, MP-H reduced total root length, root surface area, root volume, average root diameter, root-tip number, and root activity by 46.5%, 47.2%, 50.2%, 9.4%, 51.1%, and 50.9%, respectively (Figure 2A–F). MP-M also reduced total root length, root surface area, root volume, root-tip number, and root activity. Average root diameter showed a partially overlapping response: MP-M was lower than MP-L but did not differ significantly from CK or MP-H, whereas MP-H was lower than CK and MP-L. In contrast, MP-L did not significantly affect any measured root trait. Total root length, root surface area, root volume, root-tip number, and root activity were numerically higher under MP-L than under CK, but the differences were not significant.

### 3.3. Photosynthetic Pigments and Gas Exchange

**Figure 3:**
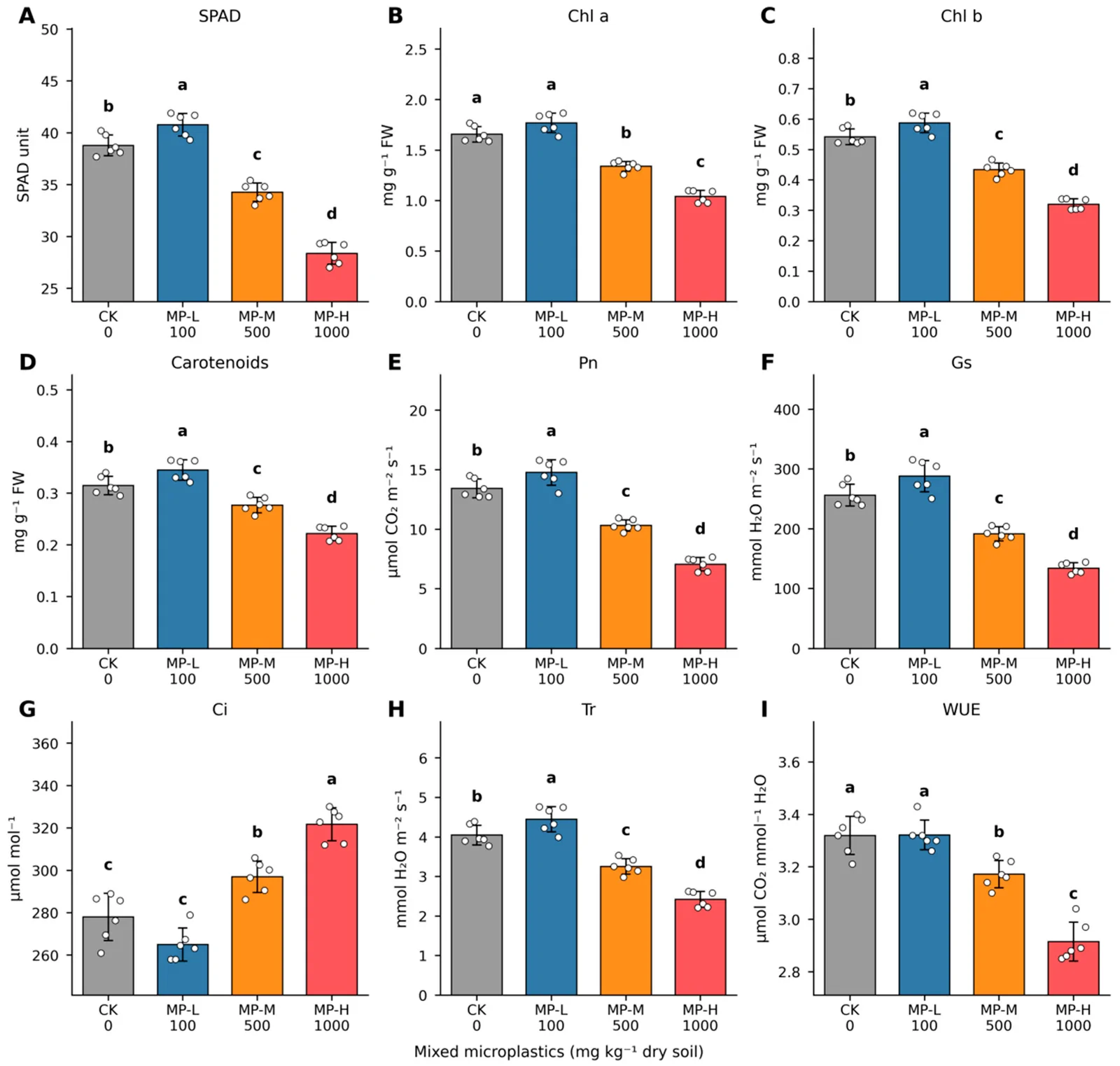
Effects of an equal-mass PVC–PE–PS microplastic mixture on leaf chlorophyll status, photosynthetic pigments, and gas-exchange traits of poplar seedlings: (**A**) SPAD value; (**B**) chlorophyll a; (**C**) chlorophyll b; (**D**) carotenoids; (**E**) net photosynthetic rate (Pn); (**F**) stomatal conductance (Gs); (**G**) intercellular CO_2_ concentration (Ci); (**H**) transpiration rate (Tr); and (**I**) instantaneous water-use efficiency (WUE). CK, MP-L, MP-M, and MP-H represent total mixture concentrations of 0, 100, 500, and 1000 mg kg^−1^ dry soil, respectively. Values are means ± SD (*n* = 6). Different lowercase letters indicate significant differences among treatments according to Tukey’s HSD test at *p* < 0.05.

Leaf pigment contents and gas-exchange performance declined under MP-M and MP-H, with the strongest responses observed under MP-H. Compared with CK, MP-H reduced SPAD value, chlorophyll a, chlorophyll b, and carotenoid contents by 26.8%, 37.2%, 41.0%, and 29.5%, respectively (Figure 3A–D). MP-H also reduced Pn, Gs, Tr, and WUE by 47.4%, 47.7%, 40.1%, and 12.2%, respectively (Figure 3E,F,H,I), whereas Ci was 15.7% higher than that in CK (Figure 3G). MP-M showed intermediate responses, with lower pigment contents, Pn, Gs, Tr, and WUE and higher Ci than CK. In contrast, MP-L produced limited and trait-specific increases: SPAD value, chlorophyll b, carotenoid content, Pn, Gs, and Tr were higher than those in CK, whereas chlorophyll a, Ci, and WUE did not differ significantly between the two treatments.

### 3.4. Chlorophyll Fluorescence Responses

**Figure 4:**
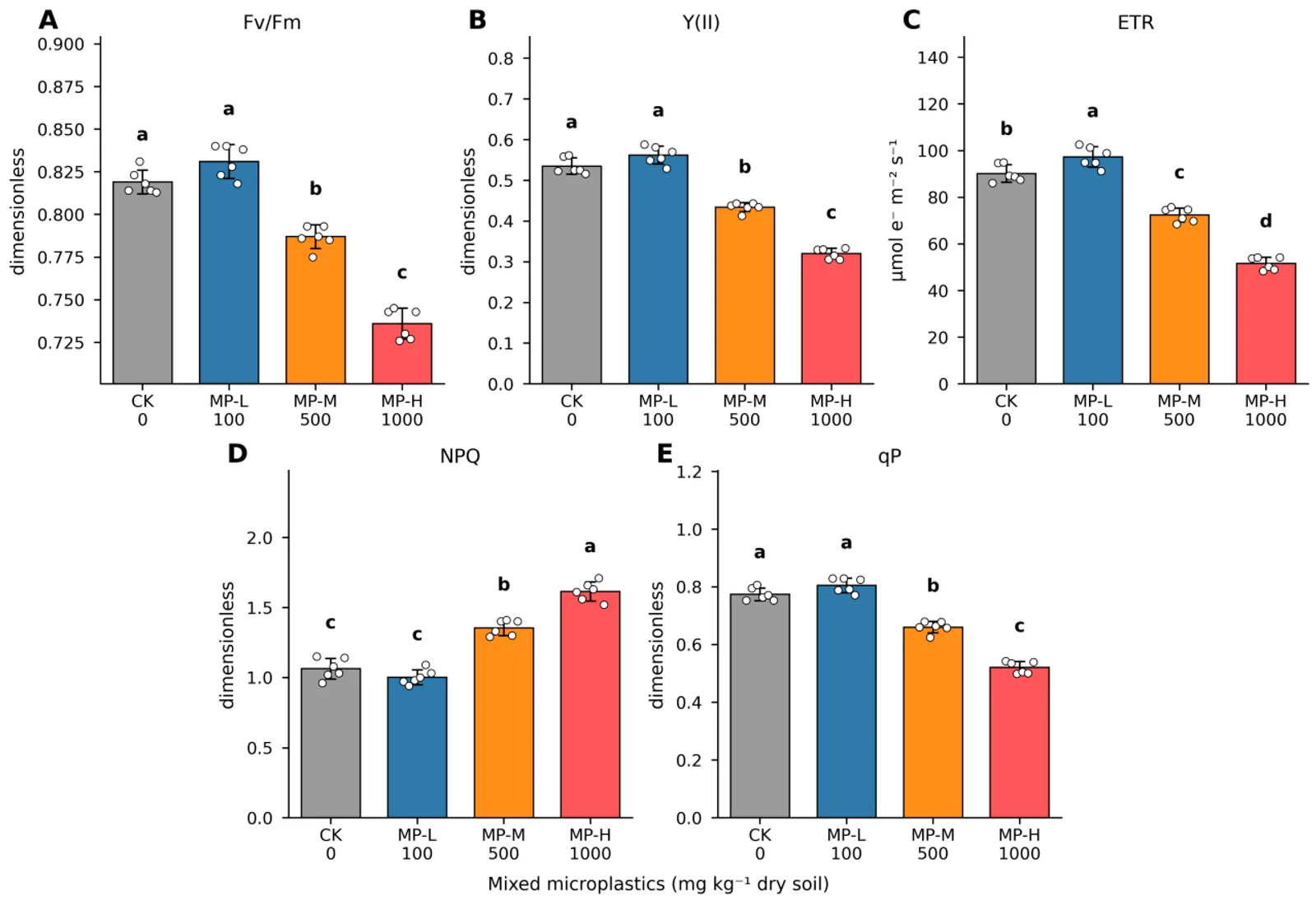
Effects of an equal-mass PVC–PE–PS microplastic mixture on the chlorophyll fluorescence parameters of poplar leaves: (**A**) maximum quantum efficiency of PSII (Fv/Fm); (**B**) effective quantum yield of PSII [Y(II)]; (**C**) electron transport rate (ETR); (**D**) non-photochemical quenching (NPQ); and (**E**) photochemical quenching coefficient (qP). CK, MP-L, MP-M, and MP-H represent total mixture concentrations of 0, 100, 500, and 1000 mg kg^−1^ dry soil, respectively. Values are means ± SD (*n* = 6). Different lowercase letters indicate significant differences among treatments according to Tukey’s HSD test at *p* < 0.05.

PSII performance declined under MP-M and MP-H, with the strongest changes observed under MP-H. Compared with CK, MP-H reduced Fv/Fm, Y(II), ETR, and qP by 10.1%, 40.2%, 42.8%, and 32.8%, respectively (Figure 4A–C,E), whereas NPQ increased by 51.9% (Figure 4D). MP-M showed intermediate changes, with Fv/Fm, Y(II), ETR, and qP reduced by 3.9%, 18.9%, 19.8%, and 14.7%, respectively, and NPQ increased by 27.5% relative to CK. In contrast, Fv/Fm, Y(II), NPQ, and qP did not differ significantly between MP-L and CK, whereas ETR was higher under MP-L.

### 3.5. Oxidative Injury and Antioxidant Enzyme Activities

**Figure 5:**
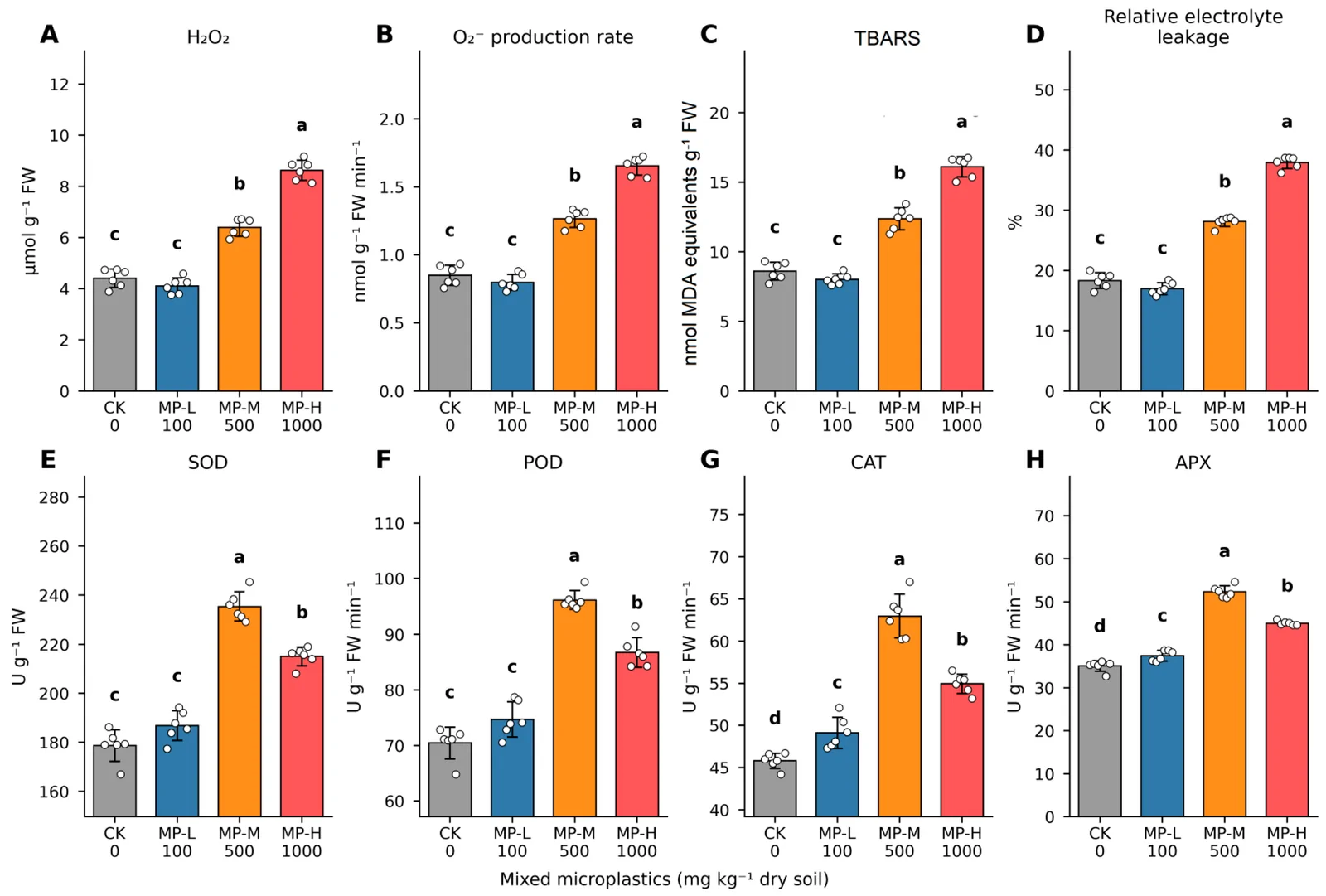
Effects of an equal-mass PVC–PE–PS microplastic mixture on oxidative-injury indicators and antioxidant enzyme activities in poplar leaves: (**A**) hydrogen peroxide (H_2_O_2_) content; (**B**) superoxide-anion (O_2_•^−^) production rate; (**C**) thiobarbituric acid-reactive substances (TBARS); (**D**) relative electrolyte leakage; (**E**) superoxide dismutase (SOD) activity; (**F**) peroxidase (POD) activity; (**G**) catalase (CAT) activity; and (**H**) ascorbate peroxidase (APX) activity. CK, MP-L, MP-M, and MP-H represent total mixture concentrations of 0, 100, 500, and 1000 mg kg^−1^ dry soil, respectively. Values are means ± SD (*n* = 6). Different lowercase letters indicate significant differences among treatments according to Tukey’s HSD test at *p* < 0.05.

Oxidative-injury indicators increased under MP-M and MP-H, with the largest increases observed under MP-H. Compared with CK, MP-H increased H_2_O_2_ content, O_2_•^−^ production rate, TBARS, and relative electrolyte leakage by 95.8%, 94.6%, 87.1%, and 106.8%, respectively (Figure 5A–D). Antioxidant enzyme activities showed a nonlinear response. The highest SOD, POD, CAT, and APX activities were observed under MP-M, with increases of 31.8%, 36.5%, 37.4%, and 49.0%, respectively, relative to CK (Figure 5E–H). Enzyme activities under MP-H were lower than those under MP-M but remained higher than those in CK. In contrast, H_2_O_2_ content, O_2_•^−^ production rate, TBARS, and relative electrolyte leakage did not differ significantly between MP-L and CK. SOD and POD activities also did not differ significantly between these two treatments, whereas CAT and APX activities were higher under MP-L.

### 3.6. Soil Chemical Properties and Soil Enzyme Activities

**Figure 6:**
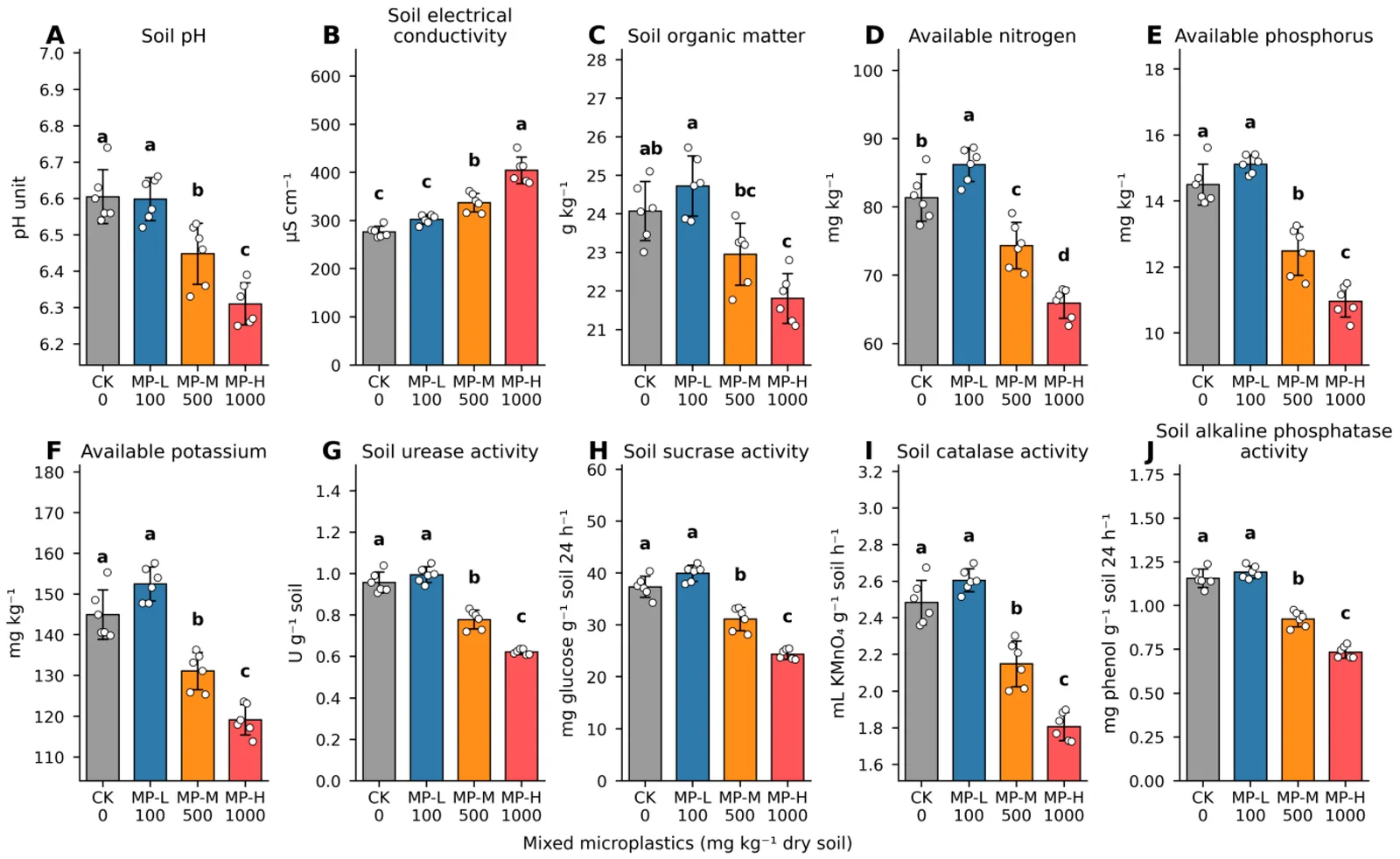
Soil chemical and enzyme responses to an equal-mass PVC–PE–PS microplastic mixture: (**A**) soil pH; (**B**) soil electrical conductivity; (**C**) soil organic matter; (**D**) available nitrogen; (**E**) available phosphorus; (**F**) available potassium; (**G**) urease activity; (**H**) sucrase activity; (**I**) soil catalase activity; and (**J**) alkaline phosphatase activity. CK, MP-L, MP-M, and MP-H represent total mixture concentrations of 0, 100, 500, and 1000 mg kg^−1^ dry soil, respectively. Values are means ± SD (*n* = 6). Different lowercase letters indicate significant differences among treatments according to Tukey’s HSD test at *p* < 0.05.

Soil variables showed the clearest treatment-associated differences under MP-M and MP-H, with the largest differences observed under MP-H. Relative to CK, soil pH under MP-H was 4.5% lower, whereas electrical conductivity was 46.3% higher (Figure 6A,B). Soil organic matter, available nitrogen, available phosphorus, and available potassium were 9.4%, 19.0%, 24.4%, and 17.8% lower, respectively (Figure 6C–F). Urease, sucrase, catalase, and alkaline phosphatase activities under MP-H were 35.0%, 34.8%, 27.2%, and 36.5% lower than those in CK, respectively (Figure 6G–J). MP-M showed intermediate treatment-associated responses. In contrast, most soil variables under MP-L did not differ significantly from CK. Soil pH, electrical conductivity, organic matter, available phosphorus, available potassium, and the four soil enzyme activities were similar between MP-L and CK, whereas available nitrogen was higher under MP-L.

## 4. Discussion

### 4.1. Growth and Root Responses to Medium and High Exposure

Medium and high exposure produced coordinated reductions in seedling growth and root-system development, with root dry mass, total root length, surface area, volume, root-tip number, and root activity generally showing stronger responses than shoot traits. This pattern indicates that the belowground compartment was particularly sensitive to the tested mixture. Reductions in root development and plant growth have also been reported in tomato and chickpea exposed to different microplastic formulations and in soil–rhizosphere–plant systems containing PS, PE, or PP, although the direction and magnitude of responses vary with polymer properties, particle size, exposure level, soil conditions, and plant species [10,21,38]. The present study extends these observations to a woody seedling exposed to a fixed equal-mass PVC–PE–PS mixture.

Several mechanisms may contribute to root responses in microplastic-containing soils. Microplastics can modify soil porosity, aggregation, aeration, water movement, and nutrient distribution [10,21,38]. Particles may also interact with root surfaces and rhizosphere processes, although the magnitude of these interactions depends on polymer characteristics, soil conditions, and plant species [10]. Therefore, the root reductions cannot be assigned to one specific process. They show that the medium and high concentrations of this particular mixture were associated with less extensive and less active root systems.

The experiment used an equal-mass mixture and did not include separate PVC, PE, and PS treatments. The contribution of each polymer and possible additive, antagonistic, or synergistic effects therefore cannot be distinguished. The conclusions should be restricted to the tested PVC–PE–PS formulation.

### 4.2. Coordinated Responses of Photosynthetic Pigments and Gas Exchange

MP-M and MP-H produced concurrent decreases in pigment content, gas exchange, and WUE. The combination of lower Pn and Gs with higher Ci indicates that stomatal restriction alone cannot explain the reduction in carbon assimilation; non-stomatal constraints, including reduced biochemical CO_2_ fixation or impaired chloroplast function, may also have contributed [14,15]. Comparable reductions in pigments and gas exchange have been reported in zucchini, tomato, and chickpea exposed to different microplastic formulations [3,6,7]. Han et al. (2026) also reported low-exposure increases and high-exposure decreases in garlic separately exposed to PVC, PE, and PS, although direct comparison is limited by differences in plant species, soil conditions, concentration ranges, and polymer-treatment design [39].

These photosynthetic changes occurred together with reductions in root surface area and root activity, which is consistent with coordinated belowground and leaf-level stress. However, plant water uptake, hydraulic traits, nutrient-uptake rates, and tissue nutrient concentrations were not measured. The present data therefore support an association between impaired root performance and photosynthetic decline rather than a demonstrated causal root-to-leaf pathway.

### 4.3. PSII Photochemistry and Energy Dissipation Under Higher Exposure

The decreases in Fv/Fm, Y(II), ETR, and qP, together with increased NPQ under MP-M and MP-H, indicate reduced PSII photochemical performance and enhanced thermal energy dissipation. Previous studies have reported microplastic-associated reductions in plant photosynthetic performance, with responses varying among particle types, sizes, concentrations, and plant species [20,21,40]. In the present study, NPQ increased under MP-H while Fv/Fm, Y(II), ETR, qP, and Pn decreased. Thus, the increase in thermal dissipation was not sufficient to maintain photochemical performance at the highest exposure.

Because chlorophyll fluorescence was measured only at the 45 d endpoint, the data cannot distinguish early reversible photoprotection from later persistent photochemical injury. Time-course and recovery measurements would be required to resolve this distinction.

### 4.4. Oxidative Injury and Antioxidant Enzyme Responses

The increased levels of H_2_O_2_, O_2_•^−^ production, TBARS, and electrolyte leakage in response to MP-M and MP-H indicated greater oxidative stress, lipid peroxidation, and membrane permeability. Such alterations were accompanied by decreased photosynthetic electron transport rates and lower growth. Redox disturbances can be induced by restricted usage of photochemical energy, which can cause excitation pressure and generation of ROS, but stresses related to root zone and nutrients might also play a role in this process [19,20,21].

The antioxidative enzyme activities were not linear. SOD, POD, CAT and APX had their maximum levels on MP-M. On MP-H, their activities were higher than in CK but below MP-M, and the ROS and membrane injury indicators were still increasing. The observed tendency shows that the response of the antioxidant enzymes was triggered during medium exposure and did not continue to rise when the highest exposure occurred.

Although SOD, POD, CAT, and APX activities were lower under MP-H than under MP-M, they remained higher than those under CK; therefore, this pattern does not demonstrate complete collapse or depletion of the antioxidant system. Plants may also rely on non-enzymatic redox buffering, the ascorbate–glutathione cycle, compatible solutes such as proline, phenylpropanoid-derived metabolites, hormone-regulated defense responses, and cellular repair processes under microplastic stress [41,42,43]. These defense pathways were not measured in the present study. The continued increases in H_2_O_2_, O_2_•^−^ production, TBARS, and electrolyte leakage under MP-H indicate that the measured enzymatic responses were insufficient to prevent oxidative and membrane injury. However, because measurements were conducted at a single 45 d endpoint and no recovery or survival assessment was performed, the present results cannot establish that the injury was irreversible or predict subsequent seedling death. Time-course and recovery experiments, together with measurements of non-enzymatic antioxidants, osmolytes, redox status, and survival, would be required to distinguish reversible acclimation from progressive injury.

### 4.5. Treatment-Associated Changes in Soil Properties and Enzyme Activities

MP-M and MP-H were associated with higher soil electrical conductivity and lower pH, available nutrients, and activities of urease, sucrase, catalase, and alkaline phosphatase. Soil organic matter was lower under MP-H than under CK, whereas MP-M did not differ significantly from CK. Because these enzymes are associated with nitrogen transformation, carbon turnover, oxidative reactions, and phosphorus cycling, respectively, the lower activities were consistent with less favorable soil biochemical conditions at the 45 d endpoint. Previous studies have shown that microplastics can alter soil aggregation, water distribution, nutrient dynamics, enzyme activities, and microbial communities, although responses vary among polymers, soils, and exposure conditions [6,7,8,10].

However, because the experiment did not include unplanted soil controls, the observed soil changes cannot be attributed exclusively to direct effects of the microplastic mixture; plant nutrient uptake and rhizosphere feedbacks may also have contributed. The soil data were therefore interpreted as treatment-associated endpoint conditions. Future studies should include parallel planted and unplanted soils, repeated sampling, and microbial measurements to distinguish direct soil effects from plant-mediated responses.

The reductions in root length, surface area, volume, root-tip number, and root activity under MP-M and MP-H were accompanied by lower Pn, Gs, Tr, and WUE and by decreases in several soil nutrient and enzyme indicators. These concurrent changes indicate coordinated belowground, leaf-level, and soil biochemical responses to the tested microplastic mixture. However, plant water uptake, hydraulic properties, tissue nutrient concentrations, rhizosphere characteristics, and soil microbial biomass or community composition were not measured. Therefore, impaired water or nutrient acquisition and microbial mediation remain possible explanations rather than demonstrated mechanisms.

Taken together, MP-M and MP-H were associated with concurrent reductions in seedling growth, root architecture and activity, photosynthetic gas exchange, PSII performance, and several soil nutrient and enzyme indicators, together with increased oxidative and membrane injury. This response pattern indicates coordinated stress across root, leaf, and soil compartments under the tested mixture. However, particle uptake, plant water and nutrient fluxes, rhizosphere microbial communities, and the temporal sequence of these responses were not measured. Therefore, the present results do not establish a single causal mechanism linking the soil, root, and leaf responses.

### 4.6. Limited and Trait-Specific Responses Under Low Exposure

MP-L did not produce a uniform stimulatory response. Most growth and root variables did not differ from CK, while leaf number, several pigment and gas-exchange traits, ETR, CAT, APX, and available soil nitrogen were higher. Oxidative-injury indicators remained similar to CK.

Low-dose increases have been reported in some microplastic–plant studies, including the PVC, PE, and PS experiment in garlic described by Han et al. [39]. The isolated increase in available soil nitrogen under MP-L may reflect a temporary shift in the balance among nitrogen mineralization, microbial immobilization, and plant uptake. However, inorganic nitrogen fractions, microbial biomass, root exudates, plant nitrogen uptake, and temporal changes in soil nitrogen were not measured, and the absence of unplanted soil controls prevents the separation of direct soil effects from plant-mediated rhizosphere responses. The specific process responsible for this increase therefore cannot be determined from the present data. This result should be interpreted as a treatment-associated endpoint response rather than evidence that MP-L enhanced soil nitrogen cycling or produced a beneficial effect.

The present MP-L results should therefore be described as limited and trait-specific responses. Additional concentrations below and between 100 and 500 mg kg^−1^, combined with repeated measurements, would be required to establish the form of the exposure–response relationship.

### 4.7. Scope, Ecological Implications, and Limitations

Recent studies indicate that microplastic effects in soil–plant systems depend strongly on polymer properties, exposure conditions, soil biological processes, and plant identity [10,11,12]. In the present experiment, the concurrent changes in root development, photosynthetic performance, oxidative status, and soil biochemical properties suggest that mixed-polymer contamination may be relevant to the early establishment of woody seedlings. From the perspective of forestry and ecological restoration, impaired root expansion and carbon assimilation could reduce seedling establishment capacity in contaminated soils. However, the present 45 d pot experiment does not establish field-scale ecological effects, and longer-term studies across different soils, climates, and poplar genotypes are required.

This study combined the measurement of whole-plant growth, root architecture, gas exchange, chlorophyll fluorescence, oxidative status, and soil endpoints. Because the two higher exposure levels exceeded most currently reported mass-based field concentrations, the magnitude of the responses observed under MP-M and MP-H should not be directly extrapolated to typical field conditions.

There are several restrictions that limit the interpretation of these results. To begin with, the experiment involved assessing only one poplar hybrid, one soil type, one exposure duration, and one identical-mass PVC-PE-PS mixture. The effects of each polymer separately and any additive, antagonistic or synergistic interactions were not able to be separated. Secondly, particle size was determined using the nominal specification of the supplier and particle recovery, aggregation, surface chemistry and transport in soil were not measured. Third, particle uptake and translocation within the plants were not measured. Therefore, the present study cannot determine whether the observed root and physiological responses resulted from direct particle–root interactions, particle uptake, or indirect changes in the soil environment.

The observed soil, root, photosynthetic, oxidative, and growth responses should therefore be interpreted as concurrent treatment-associated changes rather than a confirmed temporal or causal pathway. Time-course experiments, unplanted soil controls, polymer-specific treatments, and microbial and soil-physical analyses are required to clarify the relationships among these responses.

Because the particles were not characterized after soil incorporation and soil microbial biomass was not measured, the present results primarily describe overall treatment-associated response trends rather than particle-specific or microbial mechanisms. Future studies should combine post-mixing particle characterization with measurements of soil microbial biomass and community composition.

The use of one fixed 1:1:1 PVC–PE–PS formulation allowed comparison of responses across total exposure levels but did not permit separation of polymer-specific effects. Consequently, the observed responses should be attributed to the tested mixture as a whole and should not be interpreted as evidence of additive, synergistic, or antagonistic interactions among PVC, PE, and PS. Future studies combining single-polymer treatments with different mixture ratios will be required to resolve these interactions. Future studies combining soil enzyme assays with microbial biomass, respiration, and community analyses would help clarify the microbial processes associated with these soil responses.

## 5. Conclusions

Poplar seedlings showed concentration-dependent responses to the equal-mass PVC–PE–PS microplastic mixture. MP-L produced limited and trait-specific changes, whereas MP-M and MP-H reduced biomass accumulation, root architecture and activity, photosynthetic pigments, gas exchange, and PSII performance, with the strongest overall inhibition observed under MP-H. At this highest exposure, total dry biomass, root activity, and net photosynthetic rate decreased by 43.7%, 50.9%, and 47.4%, respectively. Under MP-H, soil electrical conductivity was higher, whereas available nutrient levels and soil enzyme activities were lower than those in CK. Among the measured ROS indicators, H_2_O_2_ showed the largest relative increase under MP-H (95.8%), whereas APX showed the strongest antioxidant enzyme response under MP-M (49.0%). However, antioxidant enzyme activities did not increase further under MP-H, where TBARS and electrolyte leakage remained elevated. These findings indicate concurrent, treatment-associated plant and soil responses to higher mixed-microplastic exposure and support the consideration of mixed-polymer contamination in future ecological risk assessment and sustainable soil management in forestry and ecological restoration settings. Future studies across additional poplar genotypes, soil types, mixture formulations, and longer exposure periods will help determine the broader relevance of these response patterns.

## Data Availability

The original contributions presented in the study are included in the article, further inquiries can be directed to the corresponding author.

## Supplementary Materials

The following supporting information can be downloaded at: https://www.mdpi.com/article/10.3390/biology15171534/s1, Table S1: Complete one-way ANOVA and Tukey HSD results for all measured variables of poplar seedlings exposed to an equal-mass PVC–PE–PS microplastic mixture.
