# Supplementary material for: Growth, Root, Photosynthetic, and Soil Responses of Poplar Seedlings to an Equal-Mass PVC–PE–PS Microplastic Mixture
Source: Biology (Basel). 2026 Sep 4;15(17):1534. doi: 10.3390/biology15171534 (PMC13565574; doi:10.3390/biology15171534)
Supplement: Supplementary file 1 [file biology-15-01534-s001.zip › biology-4458198-supplementary.pdf]

Table S1. Complete one-way ANOVA and Tukey HSD results for all measured variables of poplar seedlings exposed to an equal-mass PVC–PE–PS microplastic mixture. CK, MP-L, MP-M, and MP-H were treated as four levels of a single fixed factor. Each treatment contained six biological replicates (n = 6), with one pot containing one seedling as one independent experimental unit. F statistics are reported with numerator and denominator degrees of freedom. Tukey HSD groupings are listed in the order CK / MP-L / MP-M / MP-H; treatment means sharing at least one lowercase letter do not differ significantly at $p < 0.05$.

Trait group	Variable	Source	df	F	p	partial η²	Tukey HSD grouping (CK / MP-L / MP-M / MP-H)
Growth and biomass	Plant height	Treatment	3, 20	113.44	1.00×10^{-12}	0.944	a / a / b / c
Growth and biomass	Stem diameter	Treatment	3, 20	80.66	2.40×10^{-11}	0.924	a / a / b / c
Growth and biomass	Leaf number	Treatment	3, 20	100.24	3.20×10^{-12}	0.938	b / a / c / d
Growth and biomass	Leaf area	Treatment	3, 20	97.14	4.29×10^{-12}	0.936	a / a / b / c
Growth and biomass	Shoot fresh mass	Treatment	3, 20	102.94	2.49×10^{-12}	0.939	a / a / b / c
Growth and biomass	Root fresh mass	Treatment	3, 20	119.66	6.05×10^{-13}	0.947	a / a / b / c
Growth and biomass	Shoot dry mass	Treatment	3, 20	102.80	2.52×10^{-12}	0.939	a / a / b / c
Growth and biomass	Root dry mass	Treatment	3, 20	86.89	1.21×10^{-11}	0.929	a / a / b / c
Growth and biomass	Total dry biomass	Treatment	3, 20	101.28	2.90×10^{-12}	0.938	a / a / b / c
Growth and biomass	Root/shoot ratio	Treatment	3, 20	33.52	5.38×10^{-8}	0.834	a / a / b / c
Root traits	Total root length	Treatment	3, 20	80.81	2.36×10^{-11}	0.924	a / a / b / c
Root traits	Root surface area	Treatment	3, 20	84.31	1.60×10^{-11}	0.927	a / a / b / c
Root traits	Root volume	Treatment	3, 20	97.08	4.31×10^{-12}	0.936	a / a / b / c
Root traits	Average root diameter	Treatment	3, 20	12.79	6.84×10^{-5}	0.657	ab / a / bc / c

Trait group	Variable	Source	df	F	p	partial η^2	Tukey HSD grouping (CK / MP-L / MP-M / MP-H)
Root traits	Root tips	Treatment	3, 20	123.53	4.48×10^{-13}	0.949	a / a / b / c
Root traits	Root activity	Treatment	3, 20	134.47	2.00×10^{-13}	0.953	a / a / b / c
Photosynthetic pigments and gas exchange	SPAD	Treatment	3, 20	178.29	1.35×10^{-14}	0.964	b / a / c / d
Photosynthetic pigments and gas exchange	Chl a	Treatment	3, 20	122.48	4.85×10^{-13}	0.948	a / a / b / c
Photosynthetic pigments and gas exchange	Chl b	Treatment	3, 20	134.40	2.01×10^{-13}	0.953	b / a / c / d
Photosynthetic pigments and gas exchange	Carotenoids	Treatment	3, 20	61.97	2.64×10^{-10}	0.903	b / a / c / d
Photosynthetic pigments and gas exchange	Pn	Treatment	3, 20	122.27	4.93×10^{-13}	0.948	b / a / c / d
Photosynthetic pigments and gas exchange	Gs	Treatment	3, 20	91.61	7.40×10^{-12}	0.932	b / a / c / d
Photosynthetic pigments and gas exchange	Ci	Treatment	3, 20	48.55	2.30×10^{-9}	0.879	c / c / b / a
Photosynthetic pigments and gas exchange	Tr	Treatment	3, 20	79.52	2.74×10^{-11}	0.923	b / a / c / d
Photosynthetic pigments and gas exchange	WUE	Treatment	3, 20	52.28	1.20×10^{-9}	0.887	a / a / b / c
Chlorophyll fluorescence	Fv/Fm	Treatment	3, 20	165.95	2.68×10^{-14}	0.961	a / a / b / c

Trait group	Variable	Source	df	F	p	partial η^2	Tukey HSD grouping (CK / MP-L / MP-M / MP-H)
Chlorophyll fluorescence	Y(II)	Treatment	3, 20	252.55	4.63×10^{-16}	0.974	a / a / b / c
Chlorophyll fluorescence	ETR	Treatment	3, 20	202.51	3.94×10^{-15}	0.968	b / a / c / d
Chlorophyll fluorescence	NPQ	Treatment	3, 20	120.96	5.46×10^{-13}	0.948	c / c / b / a
Chlorophyll fluorescence	qP	Treatment	3, 20	206.50	3.26×10^{-15}	0.969	a / a / b / c
Oxidative injury and antioxidant enzymes	H_2O_2	Treatment	3, 20	209.55	2.83×10^{-15}	0.969	c / c / b / a
Oxidative injury and antioxidant enzymes	$O_2^{\bullet-}$ production rate	Treatment	3, 20	219.85	1.78×10^{-15}	0.971	c / c / b / a
Oxidative injury and antioxidant enzymes	TBARS	Treatment	3, 20	195.75	5.47×10^{-15}	0.967	c / c / b / a
Oxidative injury and antioxidant enzymes	Relative electrolyte leakage	Treatment	3, 20	522.54	3.70×10^{-19}	0.987	c / c / b / a
Oxidative injury and antioxidant enzymes	SOD	Treatment	3, 20	129.45	2.87×10^{-13}	0.951	c / c / a / b
Oxidative injury and antioxidant enzymes	POD	Treatment	3, 20	115.98	8.13×10^{-13}	0.946	c / c / a / b
Oxidative injury and antioxidant enzymes	CAT	Treatment	3, 20	111.12	1.22×10^{-12}	0.943	d / c / a / b
Oxidative injury and antioxidant enzymes	APX	Treatment	3, 20	286.31	1.36×10^{-16}	0.977	d / c / a / b

Trait group	Variable	Source	df	F	p	partial η^2	Tukey HSD grouping (CK / MP-L / MP-M / MP-H)
Soil properties and enzymes	Soil pH	Treatment	3, 20	24.34	7.01×10^{-7}	0.785	a / a / b / c
Soil properties and enzymes	Soil electrical conductivity	Treatment	3, 20	54.00	9.02×10^{-10}	0.890	c / c / b / a
Soil properties and enzymes	Soil organic matter	Treatment	3, 20	17.58	7.93×10^{-6}	0.725	ab / a / bc / c
Soil properties and enzymes	Available nitrogen	Treatment	3, 20	54.25	8.65×10^{-10}	0.891	b / a / c / d
Soil properties and enzymes	Available phosphorus	Treatment	3, 20	70.37	8.36×10^{-11}	0.913	a / a / b / c
Soil properties and enzymes	Available potassium	Treatment	3, 20	58.69	4.30×10^{-10}	0.898	a / a / b / c
Soil properties and enzymes	Soil urease activity	Treatment	3, 20	114.98	8.82×10^{-13}	0.945	a / a / b / c
Soil properties and enzymes	Soil sucrase activity	Treatment	3, 20	93.81	5.93×10^{-12}	0.934	a / a / b / c
Soil properties and enzymes	Soil catalase activity	Treatment	3, 20	77.40	3.51×10^{-11}	0.921	a / a / b / c
Soil properties and enzymes	Soil alkaline phosphatase activity	Treatment	3, 20	153.82	5.55×10^{-14}	0.958	a / a / b / c
